# Associations between Sociodemographic Factors, Lifestyle Behaviors, Pregnancy-Related Determinants, and Mediterranean Diet Adherence among Pregnant Women: The GESTAFIT Project

**DOI:** 10.3390/nu14071348

**Published:** 2022-03-24

**Authors:** Marta Flor-Alemany, Teresa Nestares, Nuria Marín Jiménez, Laura Baena-García, Virginia A. Aparicio

**Affiliations:** 1 Department of Physiology, Faculty of Pharmacy, University of Granada, 18071 Granada, Spain; floralemany@ugr.es (M.F.-A.); virginiaparicio@ugr.es (V.A.A.); 2 Institute of Nutrition and Food Technology (INYTA), Biomedical Research Centre (CIBM), University of Granada, 18016 Granada, Spain; 3 Sport and Health University Research Institute (IMUDS), Health Sciences Technology Park, 18007 Granada, Spain; nuriaproyecto@gmail.com; 4 GALENO Research Group, Department of Physical Education, Faculty of Education Sciences, University of Cádiz, 11003 Cádiz, Spain; 5 Biomedical Research and Innovation Institute of the Province of Cadiz (INiBICA), 11009 Cádiz, Spain; 6 Department of Nursing, Faculty of Health Sciences, University of Granada, 51001 Ceuta, Spain; lbaenagarcia@ugr.es

**Keywords:** diet quality, gestation, physical fitness, physical activity, Mediterranean diet

## Abstract

We examined sociodemographic factors, lifestyle behaviors, and pregnancy-related determinants associated with adherence to the Mediterranean diet (MD) during pregnancy. A total of 152 Caucasian pregnant women were included in this cross-sectional study. Dietary habits and MD adherence were assessed with a food frequency questionnaire. Physical activity (PA) levels and physical fitness (PF) components (cardiorespiratory fitness, relative muscle strength, and flexibility) were objectively measured. A clustered overall PF index was calculated. Participants with a high MD adherence were older, had a lower body mass index (BMI), spent more time in moderate–vigorous PA, had a greater overall PF, cardiorespiratory fitness, and relative muscle strength compared to participants with low MD adherence (all, *p* < 0.05). When we explored factors associated with improved MD adherence with logistic regression analysis, we found that the following factors: lower pre-pregnancy BMI (OR = 2.337; *p* = 0.026), meeting PA recommendations (OR = 2.377; *p* = 0.045), higher relative muscle strength (OR = 2.265; *p* = 0.016), and higher overall PF (OR = 5.202; *p* = 0.004) increased the chances to adhere to the MD. Older age, lower BMI, greater PF, and meeting PA recommendations were associated with higher MD adherence. These factors should be considered for a better design of educational programs and guidelines focused on improving materno–fetal health status during pregnancy.

## 1. Introduction

Inadequate maternal nutrition can adversely affect both the mother and the growing fetus [1]. Many analyses in this field have been based on a single or a few food items or nutrients [2]. Notwithstanding, epidemiological studies have underlined the importance of assessing the impact of the overall diet quality on health, emphasizing the concept of dietary patterns [3,4]. The analysis of dietary patterns has proven to be a simple and effective way to improve different health outcomes [3].

In this context, the Mediterranean diet (MD) is known to be one of the healthiest dietary patterns, which protect against the development of many diseases in all age groups [4,5,6]. Mediterranean-style diet has been associated with lower gestational weight gain and lower risk of gestational diabetes [7], lower blood pressure [8], and lower cardiometabolic risk in the adult population [5]. With this in mind, few studies have examined the potential benefits that the MD adherence could exert on maternal and fetal outcomes (considering the MD as a whole rather than focusing on the effect of its components) [6]. Regarding fetal outcomes, recent studies have shown the protective role of MD during pregnancy against excessive or insufficient fetal growth [9], preterm birth [10], neural tube defects [11], asthma and allergy [12], excessive adiposity, and other adverse metabolic markers in the offspring [13].

The identification of factors that may influence the adherence to the MD would be key in programs aimed at improving the level of adherence to this particular dietary pattern. This fact deserves special attention, taking into account studies suggesting that pregnant women are drifting away from the Mediterranean-diet-like pattern [14].

Therefore, this study aimed to evaluate the influence of sociodemographic factors (age, education, marital and working status), lifestyle behaviors (smoking habit, physical activity (PA) levels, physical fitness (PF) components), and pregnancy-related determinants (pre-pregnancy body mass index (BMI), parity, number of miscarriages, and number of children) on MD adherence during pregnancy.

## 2. Materials and Methods

### 2.1. Study Design and Participants

The complete methodology of the GEStation and FITness (GESTAFIT) project has been described elsewhere [15]. Briefly, from the 159 pregnant women who met the inclusion–exclusion criteria (Supplementary Table S1), this cross-section study included 152 pregnant women (mean age 32.9 ± 4.6 years) who had valid data in sociodemographic characteristics, lifestyle behaviors and pregnancy-related determinants, and food frequency questionnaires (Supplementary Figure S1). Written informed consent was signed by all participants.

### 2.2. Sociodemographic Factors

The evaluation procedures were carried out at the 16th gestational week (g.w.) when an initial survey (anamnesis) was performed to compile information on the sociodemographic characteristics and pregnancy-related determinants (i.e., age, number of miscarriages, parity, smoking habit, educational level, and marital and educational status).

### 2.3. Maternal Anthropometry and Body Composition

Pre-pregnancy body weight was self-reported. At the 16th g.w., height was measured using a scale (InBody R20; Biospace, Seoul, Korea) and a stadiometer (Seca 22, Hamburg, Germany). Those measurements were employed to calculate pre-pregnancy body mass index (BMI) as weight (kg) divided by squared height (m^2^).

### 2.4. Physical Activity Levels

To objectively measure PA levels at the 16th g.w., accelerometry was employed. Women were asked to wear a tri-axial accelerometer attached to their non-dominant waist (Actigraph GT3X+, Pensacola, FL, USA) for nine consecutive days. Sedentary time (min/week), moderate–vigorous physical activity (MVPA) (min/week), total PA levels (min/week), and percentage of participants who met the international PA recommendations of at least 150 min of MVPA per week were calculated [16].

### 2.5. Physical Fitness Tests

The complete PF battery employed has been previously described [15]. Briefly, the back-scratch test (as a measure of overall shoulder range of motion) was employed to assess flexibility [17]. Cardiorespiratory fitness (CRF) was assessed with the 6 min walk test along a 45.7 m rectangular course [17]. Muscle strength was evaluated by handgrip (as a measure of overall body strength) with a digital dynamometer (TKK 5101 Grip-D; Takey, Tokyo, Japan) [18]. Relative muscle strength was calculated as absolute handgrip strength divided by maternal weight at the 16th g.w. and used in the analyses as recommended to address the confounding of strength by weight status [19].

### 2.6. Clustered Physical Fitness

A clustered PF index (overall PF) was created as the mean of the z-scores ((value-mean)/(standard deviation)) of flexibility, relative muscle strength, and CRF. Higher scores indicate better PF levels.

### 2.7. Dietary Assessment

A food frequency questionnaire validated in the Spanish non-pregnant adult population was employed to assess dietary habits [20]. Although questionnaires were administered to participants at the 16th g.w. and 34th g.w. by a trained nutritionist, the present study only targeted women in the second trimester of pregnancy (from 13th to 27th g.w.). The first trimester of pregnancy is characterized in most women by morning sickness, whereas dietary habits during the second trimester of pregnancy are relatively more constant, being more representative of dietary behavior across the whole gestational period [21]. Moreover, we explored differences in MD adherence between the early second trimester of pregnancy (i.e., 16th g.w.) and the third trimester of pregnancy (i.e., 34th g.w.), and overall MD adherence remained unchanged [22]. Consequently, the dietary pattern registered at the 16th g.w. was taken as representative in this study sample.

To assess adherence to the Mediterranean dietary pattern, the Mediterranean food pattern (MFP) was employed as previously done in this study sample [22]. This dietary index was constructed with the data obtained from the food frequency questionnaire considering the intake of olive oil, fiber, fruits, vegetables, fish, cereals, meat, and alcohol. The score ranges from 5 to 40 points. However, to adapt the score to pregnant women, we did not consider alcohol consumption; thus, the maximum score for pregnant women in the present study sample ranged from 4 to 35 points. In order to avoid the discrepancies noted in the literature among the large range of cut-offs points employed, the MFP index was dichotomized using the 50th percentile, which was considered the cut-off, with participants being categorized as having low or high adherence, as performed in previous studies [22,23]. The median value of the Mediterranean diet adherence in this study sample was 21 points. Therefore, participants were classified as having a high MD adherence if they had a score of ≥21 points in the MFP index.

### 2.8. Statistical Analysis

Sociodemographic factors, lifestyle behaviors, pregnancy-related determinants, PA levels, overall PF, and individual PF components were compared between women with high MD adherence versus women with low MD adherence by Student’s *t*-test. To determine the differences among qualitative variables, a chi-square test was performed.

Univariate and multivariate logistic regression analyses were performed to explore potential sociodemographic factors, lifestyle behaviors, and pregnancy-related determinants that could be associated with MD adherence. The odds ratio (OR) with a 95% confidence interval was estimated, the level of significance was set at *p* < 0.05.

Linear regression analyses were performed to explore the association of dietary habits with overall PF and individual PF components.

Differences in dietary habits by MD diet adherence (low MD adherence vs. high MD adherence) were compared by Student’s *t*-test. Differences in dietary habits by PA recommendations (not meeting PA recommendations vs. meeting PA recommendations) were compared by Student’s *t*-test. Differences in dietary habits by smoking habit (current smoker vs. no smoker) were compared by Student’s *t*-test.

To accomplish this, the Statistical Package for Social Sciences (IBM SPSS Statistics for Windows, version 22.0, Armonk, NY, USA) was employed.

## 3. Results

Sociodemographic factors, lifestyle behaviors, and pregnancy-related determinants of the study participants by the degree of MD adherence are shown in Table 1. Participants with a high MD adherence (i.e., above median) were older (*p* = 0.022) and had a lower pre-pregnancy BMI (*p* = 0.020) compared to participants with a low MD adherence (i.e., below median). In addition, pregnant women with high MD adherence spent more time in MVPA (min/week) compared to the group with low adherence (*p* = 0.054). No differences were found regarding sedentary time and total PA (min/week) (both, *p* > 0.05). Furthermore, the group with high MD adherence had greater CRF (*p* = 0.002), relative muscle strength (*p* = 0.021), flexibility (*p* = 0.032), and overall PF (*p* < 0.001) compared to the group with low MD adherence. Differences in dietary habits by MD diet adherence (low MD adherence vs. high MD adherence) are shown in Supplementary Table S2. We found that women with a high Mediterranean diet adherence had a greater intake of whole-grain cereals (*p* < 0.001), fruits (*p* < 0.001), vegetables (*p* < 0.001), pulses (*p* = 0.002), fish (*p* < 0.001), olive oil (*p* < 0.001), and nuts (*p* < 0.001) and a lower intake of red meat and subproducts (*p* = 0.032) and sweets (*p* < 0.001).

**Figure 1:**
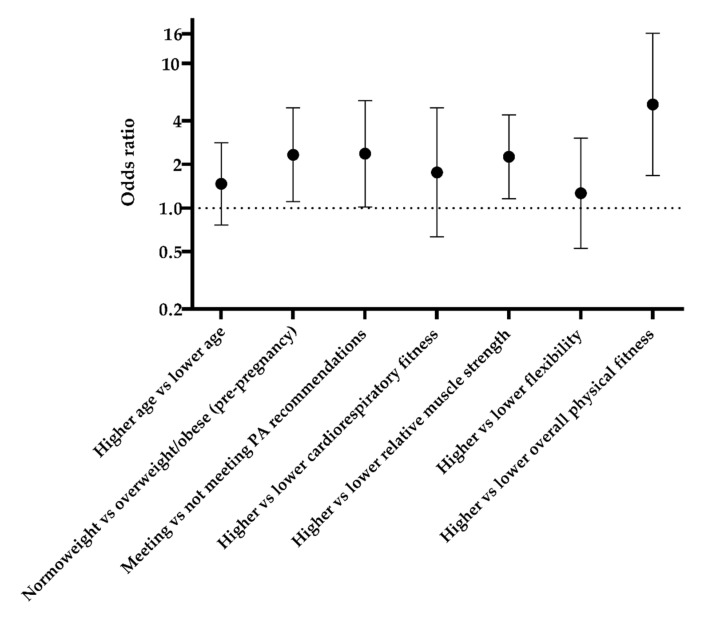
Determinants of adherence to the Mediterranean diet. PA—physical activity.

When we explored factors associated with improved MD adherence with logistic regression analysis (Figure 1), we found that the following factors: having pre-pregnancy normal weight (OR = 2.337; *p* = 0.026), meeting PA recommendations (OR = 2.377; *p* = 0.045), higher relative muscle strength (OR = 2.265; *p* = 0.016), and higher overall PF (OR = 5.202; *p* = 0.004) increased the chances to adhere to the MD.

The linear regression analysis assessing the association of dietary habits with individual PF components and overall PF is shown in Supplementary Table S3. A greater consumption of whole-grain cereals (*p* = 0.012), fruits (*p* = 0.003), and fish (*p* = 0.031) were associated with greater CRF. A higher intake of red meat and subproducts was associated with lower CRF (*p* = 0.032). Regarding relative muscle strength, a higher intake of poultry was associated with lower relative muscle strength (*p* = 0.014). No associations were found between dietary habits and flexibility (all, *p* > 0.05). In addition, a higher intake of fruits (*p* = 0.015) and vegetables (*p* = 0.004) and a lower intake of poultry (*p* = 0.041) were associated with greater overall PF.

Differences in dietary habits by PA recommendations (not meeting PA guidelines vs. meeting PA guidelines) are shown in Supplementary Table S4. Women meeting PA recommendations had a higher intake of whole-grain cereals (*p* = 0.012), lower intake of potatoes (*p* = 0.017), greater intake of fish with evidence of statistical significance (*p* = 0.053), and greater intake of nuts (*p* = 0.021) compared to their counterparts.

Differences in dietary habits by smoking habit (current smoker vs. no smoker) are shown in Supplementary Table S5. Current smokers had a lower intake of whole-grain cereals (*p* = 0.012), lower intake of fruits (*p* = 0.019), higher intake of pulses (*p* = 0.007), and higher intake of sweetened beverages (*p* = 0.024) compared to non-smokers.

## 4. Discussion

Our results suggest that a higher MD adherence was more frequent in older pregnant women and those with lower pre-pregnancy BMI. This higher adherence was also associated with healthy behaviors such as spending more time in MVPA and meeting PA recommendations, avoiding tobacco, as well as with other possible determinants of health such as greater overall PF during pregnancy.

Previous evidence [14,24] suggests that pregnant women are drifting away from the Mediterranean dietary pattern. We found that less than half of the participants (41%) of participants had a high MD adherence, which concurs with the prevalence of 33% reported by a study conducted in the same geographical area in Spanish pregnant women [25]. A recent systematic review conducted by Doyle et al. [26] has shown that dietary habits during pregnancy also depend on other health-related behaviors (with older, better educated, affluent, non-smoking, and physically active women being more likely to follow healthier dietary patterns). Therefore, this review [26] highlights the need for more studies to assess sociodemographic and pregnancy-related factors that might affect the diet during this stage. As a result, it is clinically relevant to determine factors that might be associated with this low MD adherence in this population and to identify those that might increase MD adherence.

In our study, women with higher MD adherence presented lower BMI before pregnancy, which is in agreement with previous evidence suggesting that pre-pregnancy BMI [27] was inversely associated with diet quality during pregnancy. It is important to highlight that a poorer diet quality before pregnancy may contribute to a greater pre-pregnancy BMI. As a result, it would be possible that overweight and obese pregnant women had lower diet quality and, therefore, lower MD adherence during the first trimester of pregnancy. Notwithstanding, since we do not have available data regarding dietary habits before pregnancy, we cannot verify this hypothesis. In this context, it has been suggested that the intake of individual food groups such as fruits, vegetables, and fish [28,29,30,31] remained similar during pregnancy compared to the pre-pregnancy period. In contrast, intake of red meat, bread, rice, pasta, and potatoes significantly decreased between preconception and pregnancy [28]. Usually, the overall dietary pattern does not substantially change from preconception to pregnancy periods, apart from minor changes in individual food groups [29,32].

Regarding social determinants, educational level and working status are widely employed as indicators of socioeconomic status [33]. In our study, no differences in MD adherence were found between women who had university studies and those who were working compared to their counterparts. This is in line with a previous study conducted by Maugeri et al. [34] in pregnant women, where no associations of sociodemographic characteristics with the adherence to a “prudent” dietary pattern (characterized by high intake of potatoes, raw and cooked vegetables, legumes, rice, and soup) were found. The fact that most of the participants of the present study sample had university studies and were working might explain the absence of greater associations, possibly due to our small and homogenous sample (small room for change). Notwithstanding, it has been stated that healthy dietary habits are more common among older and high-educated individuals [34]. Our results partially confirm these findings since women with a high MD adherence were older than those who had a low MD adherence. This is in agreement with previous evidence [8,13], suggesting that older women are more likely to have a better diet quality compared to younger women. The authors [8,13] attributed this difference to the fact that older women were likely to have planned pregnancies and better nutritional knowledge and, consequently, were more likely to eat more healthily to prepare for the pregnancy and to have better adherence to national guidelines and, therefore, higher diet quality.

Among the lifestyle behaviors that may exert an influence on dietary habits, smoking habit has been named as a determinant of unhealthy dietary patterns in adult pregnant and non-pregnant populations [35]. However, we did not observe a statistically significant impact of smoking habit on MD adherence during pregnancy in our study sample. This might be partially explained by the fact that the majority of pregnant women (91%) were not smokers, which might have prevented us from finding statistical differences between groups regarding MD adherence. We found that current smokers had a lower intake of whole-grain cereals and fruits and a higher intake of pulses and sweetened beverages compared to no smokers. Previous evidence [14] suggested that women who smoke during the first trimester of pregnancy consume less fruit and more sweetened beverages throughout the pregnancy and post-partum periods, and a greater amount of red and processed meat, sweet cereals, and legumes in the second trimester [14] which is in agreement with our findings.

There is a consensus that people with higher PA levels tend to present also other healthy lifestyle behaviors than their sedentary counterparts [36]. Savard et al. [37] showed that the best predictor of poorer diet quality (during the second trimester of pregnancy) was lower PA levels (assessed with the Pregnancy Physical Activity Questionnaire). In our study, participants with a high MD adherence spent more time in MVPA compared to those with a low MD adherence. This might be partially explained by the more varied diet consumed by physically active people, as previously suggested [38]. We confirmed that women meeting PA recommendations showed a greater intake of whole-grain cereals, potatoes, fish, and nuts (components of the MD) compared to their counterparts. The positive association between PA and MD adherence is noteworthy in this context, suggesting that health interventions should address adequate diet and PA levels in conjunction [25]. In addition, participants with a high MD adherence presented greater CRF, muscle strength, flexibility, and greater overall PF compared to those with low MD adherence. There is little evidence to support the association between MD adherence and/or isolated food groups and overall PF during pregnancy, but it requires special attention due to its potential maternal-fetal benefits on the prevention of adverse perinatal outcomes [39]. CRF is one of the most relevant components of PF since it is especially identified as an important marker of cardiovascular health [40]. Recent evidence suggests a strong relationship between dietary patterns and CRF [40]. In addition, this PF component is positively related to diet quality, fruit and vegetable intake and negatively associated with a meat dietary pattern (reflecting a dietary pattern with relatively high loadings of meat) [41]. Similarly, we observed that women with a high MD adherence performed better in the CRF test (walking around 40 m more) compared with women with a low MD adherence. In addition, higher consumption of whole-grain cereals, fruits, and fish was associated with greater CRF, whereas a higher intake of red meat and subproducts was associated with lower CRF. The Mediterranean dietary pattern is rich in cardio-protective nutrients; fiber; antioxidants compounds such as β-carotene, vitamin C, and vitamin E; and bioactive compounds, including monosaturated and polyunsaturated fatty acids present in plan food, fish, nuts, and extra-virgin olive oil [40,42,43]. Therefore, it seems plausible that higher adherence to this dietary pattern might be associated with greater CRF. In this sense, previous evidence has shown its effects on improving aerobic capacity [44], which might be partially explained through decreasing vasoconstriction and blood pressure [45], modulating the immune response, and reducing inflammation and oxidative stress [46].

In the same line, relative muscle strength was associated with improved MD adherence during pregnancy. This is in agreement with previous evidence [47], where an association of a healthy dietary pattern characterized by high consumption of fruits and vegetables with greater levels of muscle strength and balance was found in women during the first stage of adult life [47]. However, given the present cross-sectional design, it is not possible to determine the direction of the relationship between individual PF components, overall PF, and MD adherence. As a result, it is not clear whether a higher MD adherence is accompanied by a better PF or whether those that present an optimal PF comply with a greater MD adherence.

Limitations of the present study need to be mentioned. Firstly, a major limitation of our study is that PF tests have not been validated in pregnancy. Notwithstanding, this represents an inherent limitation of pregnancy studies, and the employed PF tests are characterized by good psychometric properties and are adaptable, viable, and safe for clinical populations [48,49,50]. Secondly, dietary patterns differ between places, populations, and cultural contexts, so a direct comparison with other non-Spanish populations and other healthy dietary patterns cannot be warranted. Regarding strengths, we include a wide range of sociodemographic, lifestyle/health-related behaviors, and pregnancy-related determinants. In addition, PA was objectively estimated through accelerometry, which is considered the “gold standard” method.

## 5. Conclusions

The current study presents evidence on specific factors influencing MD adherence during gestation. Older age, lower BMI, greater overall PF, greater CRF, muscle strength, and elements of a healthy lifestyle such as avoiding tobacco and meeting PA recommendations were associated with higher adherence to the MD. All these factors should be taken into account for a better design of specific educational programs and guidelines focused on improving health status during pregnancy.

## Figures and Tables

**Table 1:** Population characteristics by the degree of Mediterranean diet adherence.

	All Women (*n* = 152)	Low MD Adherence (*n* = 89)	High MD Adherence (*n* = 63)	*p*
Age (years)	32.9 (4.6)	32.2 (4.4)	33.9 (4.7)	0.022
Pre-pregnancy BMI (kg/m^2^) (*n* = 81 vs. 57)	24.9 (4.2)	24.9 (4.5)	23.2 (3.7)	0.020
Physical activity (*n* = 72 vs. 60)				
Sedentary time (min/day)	514.0 (91.5)	509.1 (93.0)	519.8 (90.0)	0.509
Moderate-to-vigorous physical activity (min/day)	36.4 (20.8)	33.2 (20.7)	40.3 (20.5)	0.054
Total physical activity (min/day)	423.7 (88.9)	411.8 (90.5)	437.9 (85.4)	0.092
Physical fitness				
Cardiorespiratory fitness (m) (*n* = 37 vs. 25)	605.7 (48.1)	590.8 (44.2)	627.7 (45.8)	0.002
Relative muscle strength (kg/body weight) (*n* = 89 vs. 60)	0.414 (0.08)	0.402 (0.08)	0.431 (0.07)	0.021
Flexibility (cm) (*n* = 89 vs. 61)	3.9 (6.0)	3.1 (6.2)	5.2 (5.3)	0.032
Overall physical fitness (Z-score) (*n* = 37 vs. 25)	0.1 (0.7)	−0.2 (0.6)	0.5 (0.5)	<0.001
Miscarriages *n* (%)				
No	63 (41.4)	39 (43.8)	24 (38.1)	0.480
Yes	89 (58.6)	50 (56.2)	39 (61.9)	
Parity *n* (%)				
Nullipara	91 (59.9)	52 (58.4)	39 (61.9)	0.667
Primipara	61 (40.1)	37 (41.6)	24 (38.1)	
Marital status *n* (%)				
Non-married	62 (40.8)	34 (22.4)	28 (18.4)	0.440
Married	90 (59.2)	55 (61.8)	35 (55.6)	
Educational level *n* (%)				
No university studies	62 (40.8)	41 (46.1)	21 (33.3)	0.116
University studies	90 (59.2)	48 (53.9)	42 (66.7)	
Working status *n* (%)				
Unemployed	48 (31.6)	31 (34.8)	17 (27.0)	0.305
Employed	104 (68.4)	58 (65.2)	46 (73.0)	
Smoking habit *n* (%)				
No smoking	139 (91.4)	80 (89.9)	59 (93.7)	0.414
Smoking	13 (8.6)	9 (10.1)	4 (6.3)	

Column totals not equalling the total sample size are due to missing data. *p* values for categorical variables were based on χ^2^ tests. BMI—body mass index; G.W.—gestational week; PA—physical activity.

## Data Availability

The data presented in this study are available on request from the corresponding author.

## Supplementary Materials

The following supporting information can be downloaded at: https://www.mdpi.com/article/10.3390/nu14071348/s1, Supplementary Table S1. Inclusion and exclusion criteria in the GESTAFIT project; Supplementary Figure S1. Flow diagram of the study participants; Supplementary Table S2. Differences in dietary habits by Mediterranean diet adherence (low Mediterranean diet adherence vs. high Mediterranean diet adherence). Supplementary Table S3. Linear regression analysis assessing the association of dietary habits with overall physical fitness and physical fitness components; Supplementary Table S4. Differences in dietary habits by meeting physical activity recommendations (not-meeting physical activity guidelines vs. meeting physical activity guidelines); Supplementary Table S5. Differences in dietary habits by smoking status (current smoker vs. no smoker).
