# Supplementary material for: Associations between Sociodemographic Factors, Lifestyle Behaviors, Pregnancy-Related Determinants, and Mediterranean Diet Adherence among Pregnant Women: The GESTAFIT Project
Source: Nutrients. 2022 Mar 24;14(7):1348. doi: 10.3390/nu14071348 (PMC9003033; doi:10.3390/nu14071348)
Supplement: Supplementary file 1 [file nutrients-14-01348-s001.zip › nutrients-1642799-supplementary.pdf]

Supplementary Material

Supplement to: "**Associations between sociodemographic factors, lifestyle behaviours, pregnancy-related determinants and Mediterranean diet adherence among pregnant women. The GESTAFIT project.**"

Table of contents

Supplementary Table S1. Inclusion and exclusion criteria in the GESTAFIT project.

Supplementary Figure S1. Flow diagram of the study participants.

Supplementary Table S2. Differences in dietary habits by Mediterranean diet adherence (low Mediterranean diet adherence vs. high Mediterranean diet adherence).

Supplementary Table S3. Linear regression analysis assessing the association of dietary habits with overall physical fitness and physical fitness components.

Supplementary Table S4. Differences in dietary habits by meeting physical activity recommendations (not-meeting physical activity guidelines vs. meeting physical activity guidelines).

Supplementary Table S5. Differences in dietary habits by smoking status (current smoker vs no smoker).

Supplementary Table S1. Inclusion and exclusion criteria in the GESTAFIT project.

**Inclusion criteria**
- Pregnant women aged 25-40 years old with a normal pregnancy course.
- Answering "no" to all questions on the PARmed-X for pregnancy.
- Being able to walk without assistance.
- Being able to read and write properly.
- Informed consent: Being capable and willing to provide written consent.
**Exclusion criteria**
- Having acute or terminal illness.
- Having malnutrition.
Being unable to conduct tests for assessing physical fitness or exercise during pregnancy.
- Having pregnancy risk factors (such as hypertension, type 2 diabetes, etc.).
- Having a multiple pregnancy.
- Having chromosopathy or foetal malformations.
- Having uterine growth restriction.
- Having foetal death.
- Having upper or lower extremity fracture in the past 3 months.
- Suffering neuromuscular disease or presence of drugs affecting neuromuscular function.
- Being registered in another exercise program.
- Performing more than 300 minutes of at least moderate physical activity per week.
-Being engaged in another physical exercise program
- Being unwilling either to complete the study requirements or to be randomized into the control or intervention group.

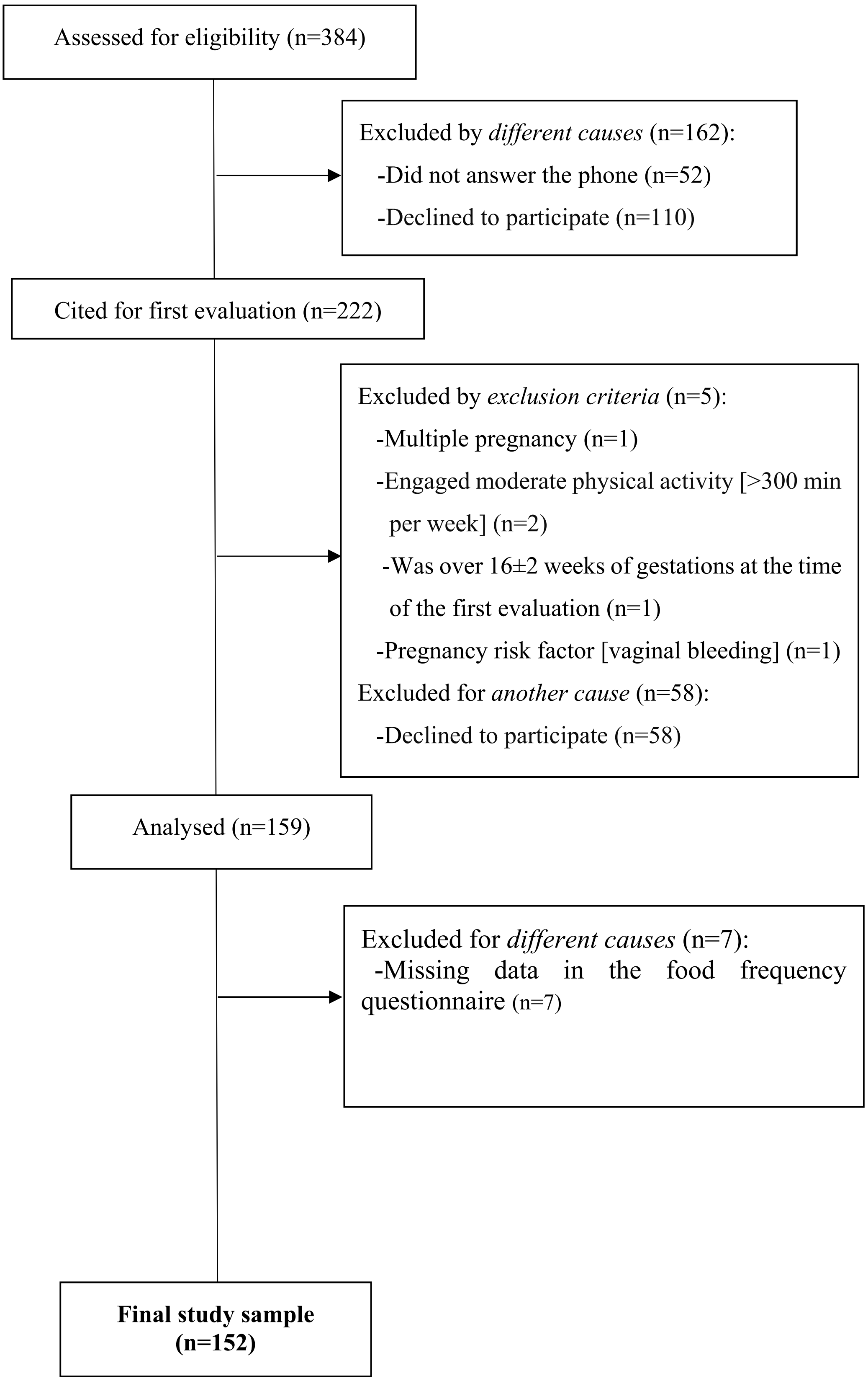

Supplementary Figure S1. Flow diagram of the study participants.

Supplementary Table S2. Differences in dietary habits by Mediterranean diet adherence (low Mediterranean diet adherence vs. high Mediterranean diet adherence).

	**Low Mediterranean diet adherence (n=89)**	**High Mediterranean diet adherence (n=63)**	***p***
Whole-grain cereals (s/week)	2.8 (4.3)	6.6 (5.4)	**<0.001**
Potatoes (s/week)	2.4 (1.3)	2.3 (1.2)	0.518
Fruits (s/week)	11.5 (5.3)	18.7 (6.5)	**<0.001**
Vegetables (s/week)	19.5 (9.1)	34.1 (11.1)	**<0.001**
Pulses (s/week)	2.5 (1.1)	3.1 (1.3)	**0.002**
Fish (s/week)	4.2 (1.9)	5.8 (2.4)	**<0.001**
Red meat and subproducts (s/week)	6.0 (3.2)	4.8 (3.6)	**0.032**
Poultry (s/week)	2.8 (1.2)	2.5 (4.5)	0.113
Dairy products (s/week)	12.9 (8.3)	12.9 (8.8)	0.958
Olive oil (s/week)	11.2 (6.6)	15.7 (6.5)	**<0.001**
Nuts (s/week)	3.3 (7.8)	7.9 (5.7)	**<0.001**
Sweets (s/week)	12.3 (7.6)	7.5 (4.1)	**<0.001**

Values shown as mean (Standard deviation). S, servings.

Supplementary Table S3. Linear regression analysis assessing the association of dietary habits with overall physical fitness and physical fitness components.

			Confidence interval 95% (B)		
	**B**	**β**	**Lower**	**Upper**	***p***
**Cardiorespiratory fitness (m) (n=62)**					
Whole-grain cereals (s/week)	2.862	0.316	0.639	5.084	**0.012**
Potatoes (s/week)	-9.129	-0.222	-19.459	1.201	0.082
Fruits (s/week)	2.190	0.366	0.752	3.629	**0.003**
Vegetables (s/week)	0.811	0.207	-0.177	1.800	0.106
Pulses (s/week)	0.215	0.006	-8.768	9.198	0.962
Fish (s/week)	5.711	0.275	0.552	10.870	**0.031**
Red meat and subproducts (s/week)	-4.111	-0.272	-7.868	-0.355	**0.032**
Poultry (s/week)	3.001	0.084	-6.214	12.217	0.517
Dairy products (s/week)	-1.313	-0.226	-2.772	0.147	0.077
Olive oil (s/week)	-0.014	-0.002	-1.746	1.719	0.987
Nuts (s/week)	1.654	0.198	-0.462	3.771	0.123
**Relative muscle strength (kg/body weight) (n=149)**					
Whole-grain cereals (s/week)	0.002	0.035	-0.006	0.009	0.671
Potatoes (s/week)	0.024	0.134	-0.005	0.052	0.102
Fruits (s/week)	0.003	0.114	-0.001	0.008	0.168
Vegetables (s/week)	0.002	0.090	-0.001	0.005	0.275
Pulses (s/week)	-0.001	-0.008	-0.031	0.028	0.922
Fish (s/week)	0.005	0.048	-0.011	0.021	0.559
Red meat and subproducts (s/week)	-0.005	-0.077	-0.016	0.006	0.350
Poultry (s/week)	-0.034	-0.200	-0.061	-0.007	**0.014**
Dairy products (s/week)	0.002	0.095	-0.002	0.007	0.251
Olive oil (s/week)	0.001	0.036	-0.004	0.006	0.667
Nuts (s/week)	0.005	0.112	-0.002	0.012	0.174
**Flexibility (cm) (n=150)**					
Whole-grain cereals (s/week)	0.071	0.062	-0.116	0.258	0.454
Potatoes (s/week)	0.121	0.026	-0.638	0.880	0.753
Fruits (s/week)	0.059	0.072	-0.074	0.192	0.384
Vegetables (s/week)	0.040	0.083	-0.038	0.117	0.312
Pulses (s/week)	-0.280	-0.058	-1.059	0.499	0.479
Fish (s/week)	0.340	0.129	-0.086	0.765	0.117
Red meat and subproducts (s/week)	-0.250	0.142	-0.534	0.034	0.084
Poultry (s/week)	-0.847	-0.176	-1.724	0.030	0.058
Dairy products (s/week)	-0.046	-0.065	-0.160	0.068	0.426
Olive oil (s/week)	0.050	0.058	-0.090	0.189	0.481
Nuts (s/week)	0.078	0.066	-0.114	0.270	0.422
**Overall physical fitness (z-score) (n=62)**					
Whole-grain cereals (s/week)	1.482	0.201	-0.384	3.348	0.117
Potatoes (s/week)	0.043	0.027	-0.378	0.464	0.838
Fruits (s/week)	3.426	0.306	0.678	6.174	**0.015**
Vegetables (s/week)	6.227	0.364	2.114	10.339	**0.004**
Pulses (s/week)	0.072	0.038	-0.424	0.569	0.772
Fish (s/week)	0.586	0.182	-0.232	1.403	0.157
Red meat and subproducts (s/week)	-0.922	-0.208	-2.040	0.196	0.104
Poultry (s/week)	-0.486	-0.260	-0.952	-0.021	**0.041**
Dairy products (s/week)	0.473	0.041	-2.502	3.448	0.752
Olive oil (s/week)	0.094	0.009	-2.481	2.670	0.942
Nuts (s/week)	1.634	0.204	-0.390	3.657	0.112

S, servings.

Supplementary Table S4. Differences in dietary habits by meeting physical activity recommendations (not-meeting physical activity guidelines vs. meeting physical activity guidelines).

	**Not meeting PA recommendations (n=103)**	**Meeting PA recommendations (n=29)**	***p***
Whole-grain cereals (s/week)	3.9 (4.6)	7.2 (6.2)	**0.012**
Potatoes (s/week)	2.5 (1.2)	1.9 (1.1)	**0.017**
Fruits (s/week)	14.2 (7.5)	16.7 (6.6)	0.109
Vegetables (s/week)	26.6 (11.9)	29.9 (11.2)	0.186
Pulses (s/week)	2.8 (1.3)	2.8 (1.2)	0.810
Fish (s/week)	4.8 (2.2)	5.7 (2.5)	**0.053**
Red meat and subproducts (s/week)	5.6 (3.5)	6.0 (3.8)	0.561
Poultry (s/week)	2.8 (1.3)	2.8 (1.8)	0.910
Dairy products (s/week)	12.4 (7.8)	13.8 (9.9)	0.409
Olive oil (s/week)	12.9 (7.1)	15.3 (6.0)	0.100
Nuts (s/week)	4.6 (4.7)	7.7 (6.3)	**0.021**

Values shown as mean (Standard deviation). PA, physical activity; S, servings.

Supplementary Table S5. Differences in dietary habits by smoking status (current smoker vs no smoker).

	**Non-smoker (n=139)**	**Current smoker (n=13)**	***p***
Whole-grain cereals (s/week)	4.6 (5.3)	1.2 (3.0)	**0.012**
Potatoes (s/week)	2.3 (1.3)	3.0 (1.3)	0.100
Fruits (s/week)	14.9 (7.2)	10.0 (6.9)	**0.019**
Vegetables (s/week)	25.5 (12.2)	29.4 (14.9)	0.273
Pulses (s/week)	2.7 (1.3)	3.3 (0.6)	**0.007**
Fish (s/week)	4.9 (2.3)	4.4 (2.0)	0.421
Red meat and subproducts (s/week)	5.5 (3.5)	6.7 (2.7)	0.159
Poultry (s/week)	2.7 (1.4)	2.9 (1.1)	0.632
Dairy products (s/week)	12.7 (8.4)	14.7 (8.9)	0.398
Olive oil (s/week)	13.2 (6.9)	11.3 (7.2)	0.338
Nuts (s/week)	5.2 (5.1)	4.4 (6.1)	0.587
Sweetened beverages (s/week)	1.6 (2.1)	3.7 (2.9)	**0.024**

Values shown as mean (Standard deviation). S, servings.
